# High expression of PALB2 predicts poor prognosis in patients with advanced breast cancer

**DOI:** 10.1002/2211-5463.12356

**Published:** 2017-12-13

**Authors:** Jingquan Li, Mian Li, Peizhan Chen, Qian Ba

**Affiliations:** ^1^ School of Public Health Shanghai Jiao Tong University School of Medicine China; ^2^ Key Laboratory of Food Safety Research Institute for Nutritional Sciences Shanghai Institutes for Biological Sciences Chinese Academy of Sciences China

**Keywords:** breast cancer, metastasis, partner and localizer of BRCA2, prognosis

## Abstract

*PALB2* mutation is associated with increased breast cancer risk; however, *PALB2* mutation is rare in sporadic breast cancer cases and little is known about PALB2 expression in breast cancer. Here, we evaluated the prognostic effects of *PALB2* with tissue microarray specimens of 117 female breast cancer patients, and determined the potential underlying mechanisms in cell models. In immunohistochemical analysis, we found increased expression of PALB2 in breast cancer tissues compared with the adjacent normal ductal epithelium (*P* < 0.001). Higher PALB2 scores were positively associated with histological grade and higher PALB2 expression was found in patients that were Her‐2 negative compared with those that were positive (*P* < 0.05). Interestingly, higher expression of PALB2 was significantly associated with poorer overall survival (*P* < 0.01) in patients with stage III or nearby lymph node metastasis (N1, N2 or N3). *In vitro* studies found that PALB2 may promote the migration and invasion of MDA‐MB‐231 cells through E‐cadherin suppression and NF‐κB activation. In conclusion, these results suggest that PALB2 expression levels may serve as a novel prognostic factor for breast cancer patients.

**Abbreviations**
DMEM: Dulbecco's modified Eagle's medium
EMT: epithelial to mesenchymal transition
ICAM1: intercellular adhesion molecule 1
IHC: immunohistochemical
IL: interleukin
NF‐κB: nuclear factor‐kappa B
PALB2: partner and localizer of BRCA2
shRNA: short hairpin RNA

Breast cancer is one of the most common life‐threatening diseases in women. Approximately 5–10% of breast cancers are caused by hereditary factors 1. Germline mutations of the *BRCA1* and *BRCA2* genes confer high risks of breast cancer. For sporadic breast cancer patients, somatic mutations are rare but allelic deletions occur at high frequency 2. BRCA1 and BRCA2 are widely expressed in breast and other tissue, where they mediate the repair of DNA double‐strand breaks by error‐free methods. *BRCA1* and *BRCA2* are widely recognized as caretaker genes for the genome and tumor suppressor genes for breast cancer. Interestingly, higher expression of BRCA2 is found in sporadic breast, ovarian, pancreatic and prostatic cancers 3, 4. A significant link between BRCA2 overexpression and tumors of histopathological grade III was observed, suggesting a role of BRCA2 in the aggressiveness of breast tumors 3. Higher BRCA2 expression is also found to be correlated with poor prognosis 5.

Partner and localizer of BRCA2 (PALB2) was first identified as colocalizing with BRCA2 in the nucleus, implicating it in the localization and stability of BRCA2 to enable its functions in homologous recombination and double‐strand break repair 6. Later, it was revealed that PALB2 acts as a linker between BRCA1 and BRCA2 in the DNA repair process 7. Epidemiological studies suggested that bialleic *PALB2* mutations are responsible for a Fanconi anemia complementation group, FANCN, which is associated with an increase in childhood cancer 8, 9. *PALB2* was also found to be a breast cancer susceptibility gene 10, 11. However, germline and somatic mutations of *PALB2* are rare in breast cancer patients, varying from 0.1% to 2.7% depending on the population 12. Little is known about *PALB2* gene expression in sporadic breast cancer. Various studies have been performed to evaluate the biological roles of PALB2 in genome stability and the DNA repair process, but whether PALB2 participates in the progression of breast cancer is unknown. In the current study, we investigated the expression of PALB2 in human breast cancer tissues and its correlation with the prognosis for breast cancer patients. The potential underlying mechanisms were also determined with *in vitro* cell models.

## Methods

### Antibodies and reagents

Polyclonal PALB2 antibody was a gift from B. Xia (The State University of New Jersey). The primary antibody for E‐cadherin was purchased from BD Biosciences (San Jose, CA, USA). Primary antibodies for Slug and Snail were purchased from Santa Cruz Biotechnology (Dallas, TX, USA). The primary antibody for β‐tubulin was purchased from Cell Signaling Technology (Danvers, MA, USA). The secondary horseradish peroxidase‐labeled goat anti‐mouse and goat anti‐rabbit antibodies were purchased from Santa Cruz Biotechnology.

### Tissue microarray and evaluation of immunostaining

A breast cancer tissue microarray was purchased from the National Engineering Center for BioChips (Shanghai, China). The tissue microarray chip contained 26 pairs of tumors and matched adjacent tissues, 91 tumor tissue samples and 12 adjacent normal tissue samples with a follow‐up time range of 8–10.8 years. All of the cases were diagnosed using histology of invasive ductal carcinomas. The expression of PALB2 in the tissues was evaluated by immunohistochemical (IHC) staining with PALB2 primary antibody (1 : 100). The staining was scored according to the staining intensity and the percentage of cells stained. The PALB2 staining intensity was graded from 0 to 3 and the percentage of cells stained was assessed from 0% to 100%. The final staining scores were calculated as staining intensity multiplied by percentage of stained cells, ranging from 0 to 3.

### Statement of human and animal rights

Informed consent was obtained from each subject, and the study methods were approved by the Ethics Committee of the Institute for Nutritional Sciences, Shanghai Institutes for Biological Sciences, Chinese Academy of Sciences. The treatment of human subjects in this study is in accordance with the ethical standards of the Declaration of Helsinki, as revised in 2008.

### Cell culture

MCF‐7, T47D, MDA‐MB‐468 and MDA‐MB‐231 cells were grown in Dulbecco's modified Eagle's medium (DMEM) supplemented with 10% fetal bovine serum and 100 U·mL^−1^ penicillin–streptomycin. All cell lines were maintained at 37 °C with 5% CO_2_.

### Plasmids, transfections and virus production

Full‐length PALB2 cDNA was cloned into pRDI lentiviral expression vector between ClaI and EcoRI site. The lentivirus was expressed in 293T cells using FuGENE6 Transfection Reagent (Roche Applied Sciences, Penzberg, Germany). MDA‐MB‐231 cells were infected with the virus in the presence of 8 μg·mL^−1^ polybrene and were selected with puromycin. BLOCK‐iT^TM^ Adenoviral RNAi Expression System (Invitrogen, Carlsbad, CA, USA) was used to transiently express short hairpin RNA (shRNA) in MDA‐MB‐231 cells. The PALB2 RNAi sequences were determined by using BLOCK‐iT^TM^ RNAi Designer (Invitrogen) and was cloned into the pENTRTM/U6 vector. The following two shRNAs were selected for human PALB2: sense 5′‐CAC CGC TTG CGA AGA TGT AGT TTC TCG AAA GAA ACT ACA TCT TCG CAA GC‐3′ and antisense 5′‐AAA AGC TTG CGA AGA TGT AGT TTC TTT CGA GAA ACT ACA TCT TCG CAA GC‐3′; and sense 5′‐CAC CGC AGC AGC AAT CTT GAC TTC TCG AAA GAA GTC AAG ATT GCT GCT GC‐3′, anti‐sense 5′‐AAA AGC AGC AGC AAT CTT GAC TTC TTT CGA GAA GTC AAG ATT GCT GCT GC‐3′.

### Cell migration and invasion assay

Cell migration was determined using a Transwell chamber with 8 μm pore size (Costar, Corning, Inc, Canton, NY, USA) in 24‐well plates. MDA‐MB‐231 Cells were transfected with small interfering RNA or overexpression vector as described above. Forty‐eight hours later, the cells were resuspended in serum‐free DMEM and seeded into the upper chamber at 1 × 10^5^ cells per well. DMEM with 10% fetal bovine serum was added into the lower chamber. After 6 h of incubation at 37 °C, the membrane was stained with 0.1% crystal violet in PBS; non‐migrated cells were then removed from the upper side of the membrane with the cotton swap. For invasion assays, the chamber membrane was initially coated with Matrigel (BD Biosciences) diluted in serum‐free medium at a ratio of 1 : 20. For the quantification of a Boyden chamber assay, cell numbers from six random fields were counted. Numbers represent mean ± SD. Statistical significance was determined by Student's *t* test (***P < 0.001 compared with control cells in each experiment).

### Western blotting

Immunoblotting was accomplished as described previously 13. In brief, equal amounts of protein extracts were separated by SDS/PAGE gels and transferred to methanol preactivated‐poly(vinylidene difluoride) membranes (Millipore, Shanghai, China). Membranes were blocked with 5% non‐fat dried milk in PBS containing 0.1% Tween 20 for 1 h at room temperature and incubated with primary antibodies against corresponding proteins overnight at 4 °C. The membranes were washed three times with PBS/0.1% Tween 20 buffer and then incubated with the appropriate secondary antibodies for 1 h at room temperature. After washing three times with PBS/0.1% Tween 20, the immunoreactive bands were visualized using the ECL Plus system (GE Healthcare, Beijing, China).

### Real‐time PCR

Total RNA was extracted from MDA‐MB‐231 cells by using Trizol reagent (Invitrogen) according to the manufacturer's instructions. One microgram of RNA was reverse‐transcribed by using the PrimeScript First Strand cDNA Synthesis Kit (Takara Bio, Otsu, Japan). The resulting cDNA samples were amplified and quantified by performing a real‐time PCR by using the primers in Table 1: The real‐time PCR was performed using a SYBR Premix Ex Taq (Takara Bio, Inc.). Normalized gene expression was calculated relative to β‐actin for all samples.

**Table 1:** The primers used in real‐time PCR

Gene	Primer
*PALB2‐F*	5′‐ACG CGT CGA CAG GCC GAA TGG TGG ATT TA‐3′
*PALB2‐R*	5′‐CAA GAT ATC GCA CAT GTA CAA ATG TGG GAA‐3′
*E‐cadherin‐F*	5′‐GAC AAC AAG CCC GAA TT‐3′
*E‐cadherin‐R*	5′‐GGA AAC TCT CTC GGT CCA‐3′
*Twist‐F*	5′‐GGA GTC CGC AGT CTT ACG AG‐3′
*Twist‐R*	5′‐TCT GGA GGA CCT GGT AGA GG‐3′
*Zeb1‐F*	5′‐ACT GCT GGG AGG ATG ACA GA‐3′
*Zeb1‐R*	5′‐ATC CTG CTT CAT CTG CCT GA‐3′
*Sip1‐F*	5′‐AGT CCA TGC GAA CTG CCA TCT GAT‐3′
*Sip1‐R*	5′‐CTG GAC CAT CTA CAG AGG CTT GTA‐3′
*Slug‐F*	5′‐ATG AGG AAT CTG GCT GCT GT‐3′
*Slug‐R*	5′‐CAG GAG AAA ATG CCT TTG GA‐3′
*Snail‐F*	5′‐ACC ACT ATG CCG CGC TCT T‐3′
*Snail‐R*	5′‐GGT CGT AGG GCT GCT GGA A‐3′
*ICAM1‐F*	5′‐AGG CCA CCC CAG AGG ACA AC‐3′
*ICAM1‐R*	5′‐CCC ATT ATG ACT GCG GCT GCT A‐3′
*IL‐6‐F*	5′‐GAA CTC CTT CTC CAC AAG CGC CTT‐3′
*IL‐6‐R*	5′‐CAA AAG ACC AGT GAT GAT TTT CAC CAG G‐3′
*IL‐8‐F*	5′‐TCT GCA GCT CTG TGT GAA GG‐3′
*IL‐8‐R*	5′‐ACT TCT CCA CAA CCC TCT GC‐3′
*GAPDH‐F*	5′‐TGC ACC ACC AAC TGC TTA GC‐3′
*GAPDH‐R*	5′‐GGC ATG GAC TGT GGT CAT GAG‐3′

### Nuclear factor‐kappa B reporter assay

Nuclear factor‐kappa B (NF‐κB) promoter activity was determined using a reporter assay. The transient transfection of control or PALB2 overexpression MDA‐MB‐231 cells was performed using FuGENE6 Transfection Reagent (Roche Applied Sciences). NF‐κB reporter plasmid and the Renilla pRL‐TK internal control vector were used for each transfection experiment. The cells were incubated for 24 h and then harvested. The luciferase activity was measured using the Dual‐Luciferase Reporter Assay System (Promega, Madison, WI, USA). The luciferase activity was adjusted for transfection efficiency by normalizing firefly luciferase activity to the *Renilla* luciferase activity generated by pRL‐TK (Promega).

### Statistical analysis

spss statistics v. 20 (IBM Corp., Armonk, NY, USA) was used for all the statistical analyses. The IHC score in each group was presented as the mean ± standard deviation, and the statistical significance of the differences for PALB2 scores between groups stratificated by disease status, ER status, PR status and Her‐2/neu status was evaluated with the a two‐independent‐samples *t* test. One‐way ANOVA was used to evaluate the differences for IHC scores between groups stratified by the various histological grades, stages and lymph node status. The prognostic impact of the PALB2 score on clinical outcome was examined by plotting overall survival curves according to the Kaplan–Meier plot method and in combination with the log‐rank test. A *P* value ≤ 0.05 was considered to be statistically significant for all statistical analyses.

## Results

### The expression of PALB2 in breast cancer patients

**Figure 1:**
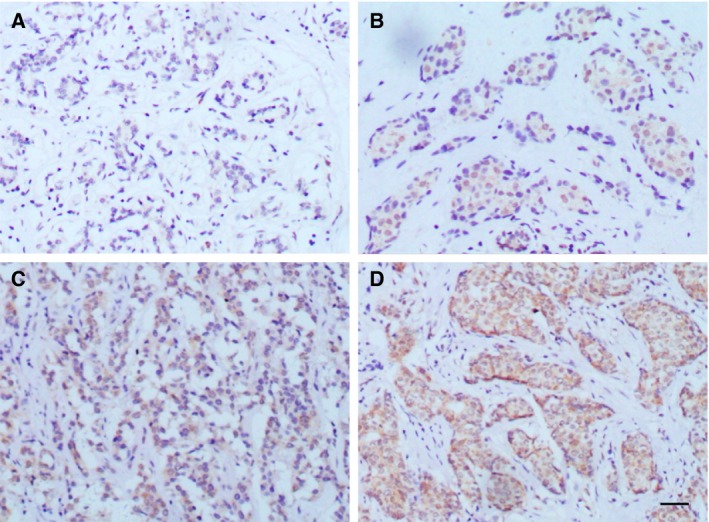
PALB2 immunohistochemistry of human breast tissue. (A) Normal breast terminal duct‐lobular unit. Very low expression was detected. (B) Invasive ductal carcinoma with PALB2 staining intensity of 1+. (C) Invasive ductal carcinoma with PALB2 staining intensity of 2+. (D) Invasive ductal carcinoma with PALB2 staining intensity of 3+. Bars = 50 μm.

**Figure 2:**
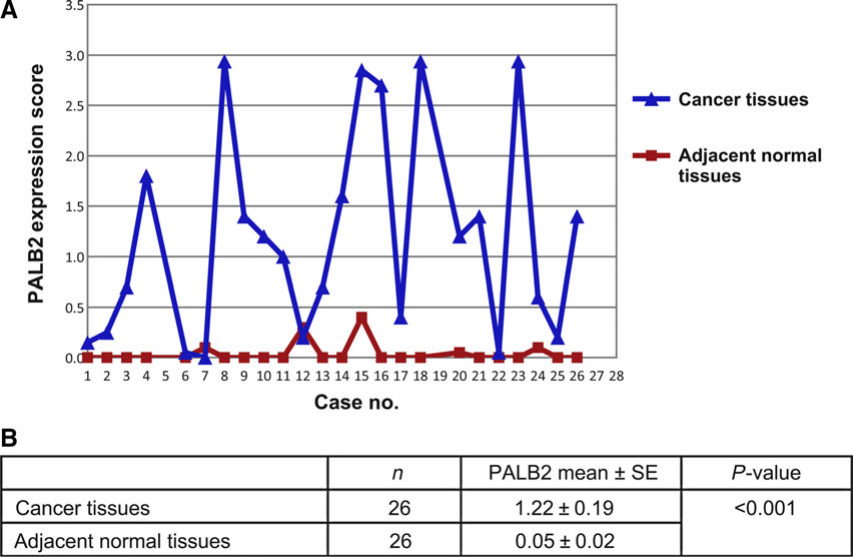
Increased expression of PALB2 in human breast cancers. (A) PALB2 scores in 26 pairs of breast samples. (B) Statistical results of PALB2 expression in breast carcinoma and adjacent normal tissues.

The expression of PALB2 in the tissue array specimens was determined using IHC staining density scores (Fig. 1). The array included the tumor tissue samples and their corresponding adjacent normal tissue samples from 26 female breast cancer patients. All of the patients were diagnosed with invasive ductal carcinoma. The mean age of the patients was 54.3 years (range 36–77 years). No PALB2 or very low PALB2 expression level (all IHC score < 0.5) was observed in the adjacent normal tissues, while a significantly higher PALB2 score was detected in breast carcinoma samples (mean IHC score = 1.22, *P* < 0.001, Fig. 2). A total of 117 breast carcinoma specimens were available for analysis of PALB2 IHC staining. Higher PALB2 expression level was detected in patients with higher tumor histological grade (II–III and III) or with Her‐2 negative status (Table 2). No significant difference of PALB2 IHC score was detected in breast cancer patients with different stage (higher *vs* lower), lymph nodes status (positive *vs* negative), ER status (positive *vs* negative) and PR status (positive *vs* negative).

**Table 2:** The clinicopathological features of 117 cases and their correlations with PALB2 scores

	Number of cases (%)	PALB2 mean	*P*‐value
Histological grade			
I, I–II	39 (33.33)	0.868	0.020
II	74 (63.25)	1.200	
II–III, III	4 (3.42)	1.875	
Stage			
I	6 (5.31)	0.892	0.346
II	69 (61.06)	1.211	
III	38 (33.63)	0.996	
Lymph node status			
N0	42 (37.17)	1.358	0.099
N1	36 (31.86)	0.9	
N2	27 (23.89)	1.041	
N3	8 (7.08)	1.155	
Estrogen receptor			
Positive	74 (63.25)	1.130	0.641
Negative	43 (36.75)	1.083	
Progesterone receptor			
Positive	65 (55.56)	1.075	0.449
Negative	52 (44.44)	1.159	
Her‐2			
Positive	30 (25.64)	0.905	0.022
Negative	87 (74.36)	1.184	

### Higher PALB2 expression was associated with poorer overall survival time in advanced breast cancer patients

**Figure 3:**
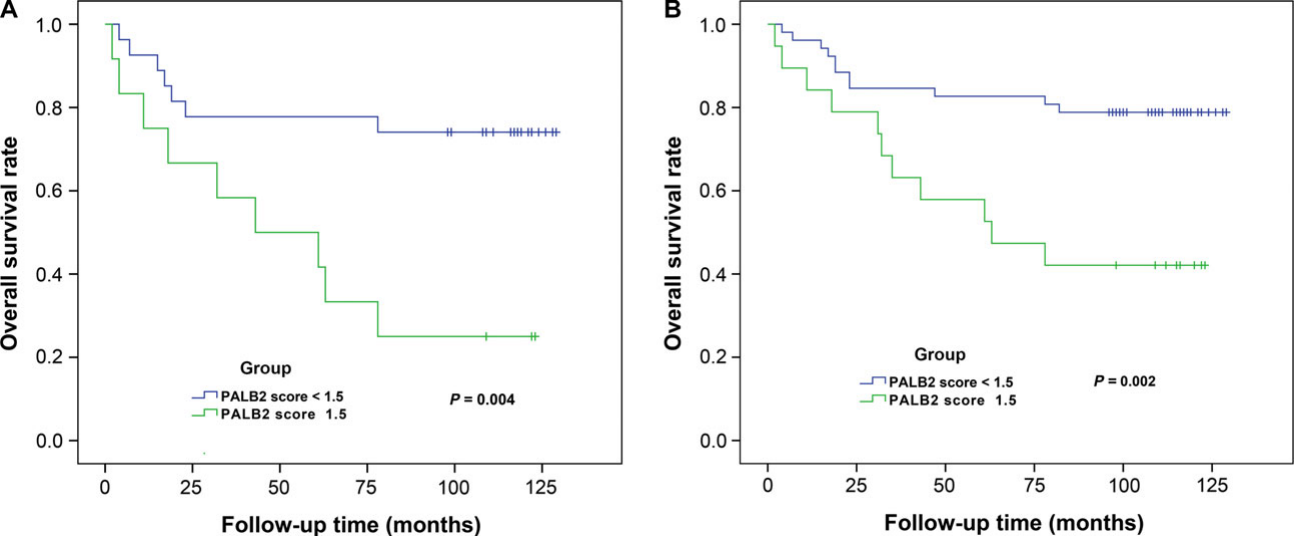
PALB2 expression is associated with overall survival of breast cancer patients. (A) Kaplan–Meier survival curves show the overall survival of 38 stage III breast cancer patients with high PALB2 score (≥ 1.5) or low PALB2 score (< 1.5) invasive ductal carcinoma. (B) Kaplan–Meier survival curves show the overall survival of 71 breast cancer patients having nearby lymph nodes involved (N1, N2 or N3) with high PALB2 score (≥ 1.5) or low PALB2 score (< 1.5) invasive ductal carcinoma.

The follow‐up time of all patients ranged from 2 to 129 months (median, 80.2 months). We evaluated the association between the IHC score and the survival of breast cancer patients. No significant association was found for the overall survival time of the patients and the PALB2 IHC score. However, among 38 patients with stage III breast cancer, higher PALB2 expression (IHC score ≥ 1.5, 11 samples) was found to be associated with poorer overall survival compared with those with lower PALB2 expression (IHC score < 1.5, 27 samples) as suggested by the log‐rank test for the Kaplan–Meier plot of the overall survival time for cancer (*P* = 0.004, Fig. 3A). In addition, for the 71 breast cancer patients with nearby lymph nodes involved (N1, N2 or N3), high PALB2 score (IHC score ≥ 1.5) was significantly associated with poorer overall survival (*P* = 0.002, Fig. 3B) as suggested by the log‐rank test. These results indicated that PALB2 might act as a novel biomarker for the prognosis of the breast cancer patients and that PALB2 might participant in the progression of breast cancer.

### PALB2 promoted MDA‐MB‐231 cell migration and invasion

**Figure 4:**
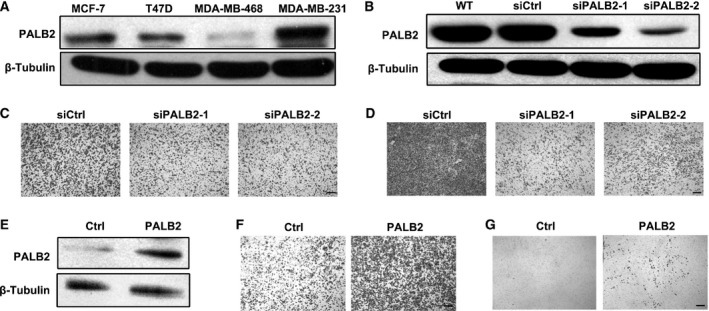
PALB2 promotes MDA‐MB‐231 cell migration and invasion with little effect on cell proliferation. (A) Western blot analysis of PALB2 expression in four different human breast tumor cell lines. (B) Western blot analysis of lysates from control and sh‐PALB2 MDA‐MB‐231 cells following retroviral‐mediated PALB2 knock‐down. (C) Knockdown of PALB2 decreased the migration activity of MDA‐MB‐231 cells. (D) Knockdown of PALB2 decreased the invasion activity of MDA‐MB‐231 cells. (E) Western blot analysis of lysates from control vector and pRDI‐PALB2 stable overexpression MDA‐MB‐231 cells. (F) Overexpression of PALB2 promoted MDA‐MB‐231 cell migration. (G) Overexpression of PALB2 promoted MDA‐MB‐231 cell invasion. Bars = 150 μm.

**Figure 5:**
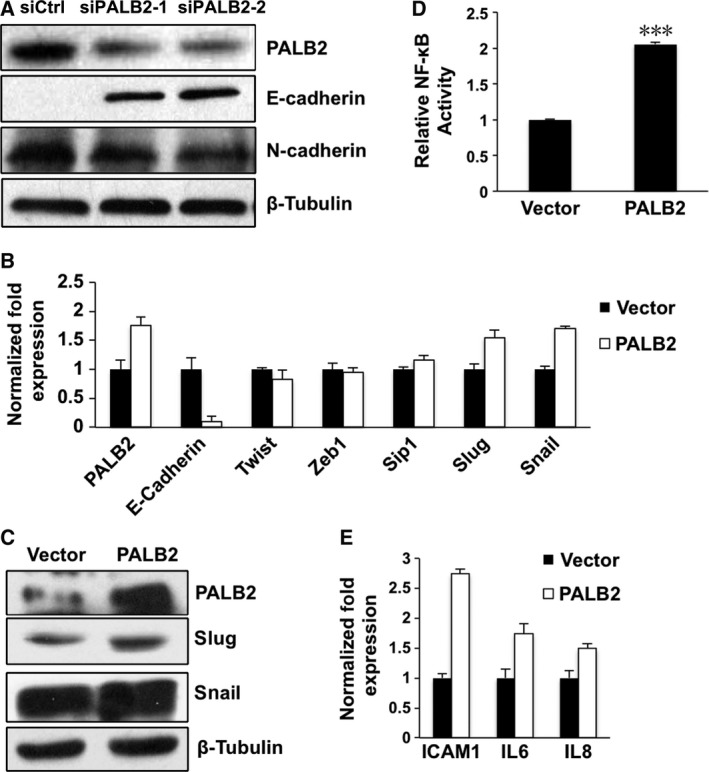
Effects of PALB2 on the transcription of E‐cadherin and NF‐κB activation in MDA‐MB‐231 cells. (A) Western blot analysis of E‐cadherin and N‐cadherin expression in control or sh‐PALB2 MDA‐MB‐231 cells. (B) Real‐time PCR analysis of the expression of E‐cadherin and related transcription factors following PALB2 overexpression. (C) Western blot analysis of the expression of the transcription factors in control or PALB2 stable overexpression MDA‐MB‐231 cells. (D) NF‐κB reporter assay showing NF‐κB activation following PALB2 overexpression. (E) Real‐time PCR analysis showing increased expression of ICAM1, IL6 and IL8.

We examined the PALB2 expression level in several human breast cancer cell lines, namely MCF‐7, T47D, MDA‐MB‐468 and MDA‐MB‐231, by western blot. High expression of PALB2 was found in MDA‐MB‐231 cells while lower expression was found in MCF‐7, T47D and MDA‐MB‐468 cells, which exhibit low invasive capacity compared with MDA‐MB‐231 (Fig. 4A). To investigate the possible effects of PALB2 in breast cancer cell invasion, *PALB2* was knocked down in MDA‐MB‐231 cells with two different shRNAs (Fig. 4B). Decreased expression of PALB2 significantly inhibited cell migration and invasion in Boyden chambers (Fig. 4C–G). An epithelial to mesenchymal transition (EMT) usually occurs during cancer progression. Downregulation of E‐cadherin expression is a key initiating event in EMT 14 and transcription factors that repress E‐cadherin have often been defined as inducers of EMT. The naïve MDA‐MB‐231 cell is negative for E‐cadherin expression. However, a significant increase in E‐cadherin was detected following *PALB2* knockdown. In contrast, the expression of N‐cadherin was decreased when the PALB2 level was reduced (Fig. 5A). In the PALB2 overexpression cell line, the transcription of E‐cadherin was significantly inhibited. The transcriptional repressors that are known to downregulate E‐cadherin expression, Slug and Snail, were activated (Fig. 5B); higher expression of Slug and Snail was also seen by western blotting following PALB2 up‐regulation (Fig. 5C). In addition, we also noticed that NF‐κB was activated by PALB2 overexpression (Fig. 5D), which was also confirmed by the enhanced transcription of *intercellular adhesion molecule 1* (*ICAM1*), and *interleukins 6* and *8* (*IL6* and *IL8*; Fig. 5E).

## Discussion

In the present study, PALB2 was found to participate in human breast cancer progression. We demonstrated for the first time that higher expression of PALB2 was correlated with poor prognosis for advanced breast cancer patients. *In vitro* studies suggested that overexpression of PALB2 promotes the migration and invasion of MDA‐MB‐231 cells through transcriptional suppression of E‐cadherin. Knockdown of *PALB2* inhibits the migration and invasion ability of the breast cancer cells through increased E‐cadherin expression level and decreased N‐cadherin expression level. These data suggest that PALB2 may be involved in the EMT process that promotes the invasion and metastasis of breast tumor, leading to the poor outcomes of breast cancer patients.

PALB2 colocalized with BRCA1 and BRCA2 and they acted together in error‐free homologous recombination repair 15. As dysfunction of BRCA1 and BRCA2 promotes carcinogenesis through promoting genomic instability 16, *PALB2* has been hypothesized to be a tumor suppressor gene. Unexpectedly, we have found that the expression of PALB2 is significantly up‐regulated in cancerous tissues compared with adjacent normal samples. Interestingly, a significant link was observed between PALB2 overexpression and tumors of histopathological grade III (*P* = 0.02), suggesting that overexpression of PALB2 plays a role in the aggressiveness of breast tumors. Possible explanations include that nuclear pleomorphism of the cancer cells induces PALB2 expression to promote DNA repair, or PALB2 expression could be induced by cellular proliferation.

Few reports have evaluated the relationship between PALB2 expression and cancer patient outcomes. Herein, we have found that high PALB2 expression was significantly associated with poor overall survival in stage III breast cancer patients and in patients with lymph node metastasis involved (N1, N2 or N3). However, no significant association was noticed in early and low‐risk breast cancer patients, which may be due to the smaller sample size of the patients. These results suggest that PALB2 expression level could be an independent prognostic factor for breast cancer patients.

Higher expression of PALB2 was also found in the highly metastatic breast cancer cell line MDA‐MB‐231 compared with low invasion capacity cell lines such as MCF‐7, T47D and MDA‐MB‐468. MDA‐MB‐231 cells are of mesenchyme origin and triple‐negative phenotype 17. The *in vitro* studies suggested that *PALB2* knockdown will decrease the migration ability of the cells and overexpression of PALB2 increase the migration or invasion ability of the cancer cells. Considering that PALB2 mutants may exist in MDA‐MB‐231 cells, and such mutants may be attributed to the oncogene activity of *PALB2*, the full length of *PALB2* in MDA‐MB‐231 cells was sequenced. No mutation was found in the *PALB2* coding region. Complete loss of E‐cadherin protein expression has been found in 84% of invasive lobular breast carcinomas 18. The re‐expression of E‐cadherin together with decreased expression of N‐cadherin was found in the mesenchyme‐like MDA‐MB‐231 cells following down‐regulation of PALB2 expression. On the contrary, the mRNA level of E‐cadherin was greatly decreased when PALB2 was overexpressed. As EMT regulators such as Slug, Snail and NF‐κB are important regulators for E‐cadherin, we evaluated the expression level of E‐cadherin and the transcription factors and found that PALB2 may affect the EMT of breast cancer cells. Our finding was consistent with previous reports that high BRCA2 mRNA levels are associated with an aggressive phenotype of sporadic breast cancer 3. High BRCA2 mRNA was significantly associated with poor prognosis of breast cancer patients 19. The high expression of PALB2 may potentially promote the function of BRCA2 to cause or enhance the progress of breast cancer. PALB2 may also act independently from BRCA2 to regulate the metastasis of the breast cancer cells. The roles of PALB2 in breast cancer progression need further investigation.

In conclusion, we firstly evaluated the expression of PALB2 in sporadic breast cancer and the association between PALB2 expression and prognosis of the breast cancer patients. High expression of PALB2 was found in breast tumors and was significantly associated with enhanced aggressive phenotypes. PALB2 high expression was also found to predict poor overall survival in advanced breast cancer patients. These results suggest for the first time that PALB2 expression level might serve as a clinically useful prognostic factor in breast cancer patients.

## Author contributions

JL and ML carried out the experiment. PC analyzed and discussed the data. JL and QB wrote the manuscript.
